# Matrix-Assisted Cell Transplantation for the Treatment of Limbal Stem Cell Deficiency in a Rabbit Model

**DOI:** 10.3390/biomedicines12010101

**Published:** 2024-01-03

**Authors:** Yang Yu, Andrey Yurevich Andreev, Olga Sergeevna Rogovaya, Anastasia Mikhailovna Subbot, Sergey Petrovich Domogatsky, Sergey Eduardovich Avetisov, Ekaterina Andreevna Vorotelyak, Egor Olegovich Osidak

**Affiliations:** 1 Department of Eye Diseases, I.M. Sechenov First Moscow State Medical University, 8-2, Trubetskaya Street, 119991 Moscow, Russia; 2 Department of Pathologies of Optical Medium of the Eye, Krasnov Research Institute of Eye Diseases, 11A Rossolimo St., 119021 Moscow, Russia; 3 R&D Department, Imtek Ltd., 3rd Cherepkovskaya 15A, 121552 Moscow, Russia; spdomo@gmail.com; 4 Laboratory of Cell Biology, Koltzov Institute of Developmental Biology Russian Academy of Science, 26, Vavilova St., 119334 Moscow, Russia; 5 Laboratory of Basic Research in Ophthalmology, Krasnov Research Institute of Eye Diseases, 11A Rossolimo St., 119021 Moscow, Russia; 6 Laboratory of Immunochemistry, FSBI National Medical Research Centre of Cardiology Name after Academician E.I. Chazov of the Ministry of Health of the Russian Federation, Academika Chazova St., 15A, 121552 Moscow, Russia; 7 Laboratory of Cellular Hemostasis and Thrombosis, Dmitry Rogachev National Medical Research Center of Paediatric Haematology, Oncology and Immunology, Samora Machel St., 1, 117997 Moscow, Russia

**Keywords:** limbal stem cell deficiency, collagen, limbal stem cell, matrix-assisted cell transplantation

## Abstract

With the development of regenerative medicine in ophthalmology, the identification of cells with high proliferative potential in the limbal area has attracted the attention of ophthalmologists and offered a new option for treatment in clinical practice. Limbal stem cell deficiency (LSCD) is an identified eye disease with a difficult and negative outcome, for which the traditional treatment is keratoplasty. This study sought to evaluate the efficacy of matrix-assisted cell transplantation consisting of in vitro-cultured autologous limbal stem cells (LSCs) and type I collagen for the treatment of LSCD in rabbits. LSCD was induced in 10 rabbits by a combination of mechanical limbectomy and alkali burns. Cells were cultured on a plate for 14 days before being transferred to a collagen-based matrix for another 7 days. Rabbits were divided into two groups as follows: the experimental group (five rabbits) received matrix-assisted cell transplantation, while the control group (five rabbits) received only conservative therapy with anti-inflammatory eye drops. During the postoperative period, all rabbits were examined using slit-lamp biomicroscopy with photo-registration and fluorescent staining, impression cytology and anterior segment optical coherence tomography (AS-OCT). Rabbits were euthanized at 30 and 120 days, and their corneas were processed for histology and immunohistochemistry. As a consequence, rabbits in the experimental group demonstrated the restoration of the corneal epithelium and transparency without epithelial defects. Moreover, goblet cells were absent in the central zone of the corneal epithelium. In conclusion, our new method of treatment enhanced the corneal surface and is an effective method of treatment for LSCD in rabbits.

## 1. Introduction

Limbal stem cell deficiency (LSCD) is one of the leading causes of blindness in all corneal diseases, manifesting as corneal opacity, conjunctival and vascular ingrowth into the cornea, and decreased visual acuity. It is known that the main causes of LSCD include non-immunological factors affecting the limbal area, such as chemical, thermal, mechanical iatrogenic and autoimmune diseases, including Stevens–Johnson syndrome, mucous membrane pemphigoid and ocular surface allergic diseases. Furthermore, genetic disorder plays a significant role in the development of LSCD in regard to a number of hereditary eye diseases, such as congenital aniridia, autoimmune polyendocrinopathy candidiasis ectodermal dystrophy (APECED), etc. [1]. The pathogenesis of LSCD is based on injury to limbal stem cells (LSCs) and/or disruption of their microenvironment (limbal niche).

The limbus is a ribbon-shaped translucent portion that transitions from the transparent cornea to the opaque sclera and serves as an anatomical and functional barrier in the cornea against the ingrowth of the conjunctival epithelium and vessels. The limbal Vogt palisade folds on the basal layer contain limbal stem cells, whose function is regulated by limbal mesenchymal stem cells (MSCs) [2,3,4,5]. MSCs maintain the normal homeostasis of the corneal epithelium and restore its integrity in response to injury. The choice of therapeutic or surgical treatment strategies is determined by the severity and form of the disease, and treatment consists of restoring the function of the limbal niche and/or the anatomical structure of the limbus, including the integrity of the corneal epithelium and the replenishment of cell quantities and composition.

Conservative therapy includes the use of bandage contact lenses, lubricating eye drops, anti-inflammatory eye drops and blood-derived eye drops [6,7,8,9]. However, due to their ineffectiveness and the reduced quality of life caused by frequent instillations and exacerbations, a new therapeutic approach is required. Currently, several surgical methods for the treatment of LSCD have been developed, including tissue transplantation or transplantation with new biomaterials containing a population of viable LSCs [10].

Limbal allograft and autograft transplantations are distinguished by the source of donor tissue. A large-sized graft is required, and the most significant disadvantage of any limbal transplantation is the high risk of iatrogenic LSCD development in the healthy eye. Currently, limbal autograft transplantation has been well studied and investigated, with a high success rate of 81% in restoring corneal anatomical integrity and improving visual acuity by 74.4% [11]. However, a number of disadvantages associated with this method limit its clinical utility in the treatment of LSCD. These include contraindications in bilateral LSCD, as well as a high risk of iatrogenic LSCD in the contralateral eye, especially when a large section of tissue is taken for transplantation [11,12,13]. This problem can be solved by modern cell technology, which allows the use of small tissue sections to produce the necessary number of limbal cells capable of curing LSCD if they are successfully transplanted [14,15,16]. However, when cells are transplanted as a suspension, there is a problem with the localization and survival of cells at the injection site, which can be solved by using a large number of LSCs [17].

Modern methods of cultivation allow for the accumulation of the required number of cells, but it is necessary to minimize the time that LSCs are present in culture in vitro due to the risk of depletion of their population.

Various hydrogels are used to ensure cell localization and survival at the injection site, allowing for a reduction in the number of cells required for treatment. Hydrogels used for cell transplantation must have the following minimum essential properties: (1) they must be biocompatible; (2) they must support high cell survival and functionality in vitro and in vivo; and (3) they must be suitable for transplantation using standard surgical methods.

In our previous study [18], we demonstrated that collagen hydrogels prepared from a concentrated solution of type I collagen met the majority of these criteria. However, the ability of cells within such a carrier to exert a clinically relevant effect in vivo has not been investigated.

Thus, the goal of this study was to create a method of limbal stem cell transplantation with a high-density collagen hydrogel and to evaluate the results of transplanting this tissue-engineered construct into experimental animals with a previously created model of LSCD.

## 2. Materials and Methods

### 2.1. Animals

In the experiment, 12 female *Chinchilla* rabbits with an average age of 6 months and a weight of 3.5 kg were used. All experiments were carried out in compliance with Directive 2010/63/EU and the Research Institute of Eye Diseases Animal Care and Use Committee guidelines, and the study was approved by the aforementioned institution’s review board.

All animal surgeries were performed in the operating room under combined anesthesia and in accordance with aseptic protocols. Preoperative preparation included the treatment of the operating field using the Pirogov–Grossich–Filonchikov technique, blepharostat installation, local anesthesia in the form of subconjunctival or subtenon injection with 0.5 mL solution of lidocaine 2% and epibulbar instillation with Alcaine (proxymetacaine 0.5%) twice. In addition, 0.4 mL of Zoletil 50 (tiletamine 25 mg/mL and zolazepam 25 mg/mL) and 0.7 mL of xylazine 2% were injected intramuscularly.

### 2.2. Matrix-Assisted Cell Transplantation

#### 2.2.1. Scaffold

Based on preliminary in vitro studies, a Viscoll collagen membrane (Imtek Ltd., Moscow, Russia) composed of type I collagen with a concentration of 10 mg/mL was selected as the scaffold for the cells [19].

#### 2.2.2. Cells

1. Obtaining a biopsy specimen.

A conjunctival incision was made along the limbus from 8 to 14 h to obtain a limbal biopsy. The conjunctiva and tenon sheath were separated, and hemostasis was performed. The tissue fragment was excised 1 mm from the corneal and conjunctival sides with a sharp ophthalmic blade. Two knotted sutures were used to close the wound, and at the end of the operation, 0.3 mL of 0.4% dexamethasone was injected.

2. Cell isolation and cultivation.

The removed tissue was cut into 2 × 4 mm pieces and thoroughly washed with Hanks’ solution (PanEco, Moscow, Russia) containing antibiotics (gentamicin 0.16 mg/mL and streptomycin 1 mg/mL) and amphotericin B (5 mg/mL). The biopsy specimen was then placed in 2% dispase solution (Gibco, USA) in DMEM (PanEco, Moscow, Russia) and fermented for 1 h at 37 °C. After the first stage of fermentation under a binocular microscope, a portion of the sclera was partially detached from the biopsy with forceps. The separated epithelium with remaining sclera was cut into small pieces 1–2 mm in size and washed with EDTA solution (PanEco, Moscow, Russia), followed by a secondary fermentation with 0.25% trypsin solution in EDTA (PanEco, Moscow, Russia) for 20 min at 37 °C. Trypsin was inactivated by DMEM/F12 medium (PanEco, Moscow, Russia) containing serum (10% fetal bovine serum (FBS)), after which limbal fragments were intensively pipetted, followed by centrifugation at 400× *g* for 6 min. The supernatant was drained, and the residue with undissolved limbal fragments and individual cell conglomerates was resuspended in DMEM/F12 nutrient medium (PanEco, Moscow, Russia) with the addition of 10% fetal calf serum (HyClone, Logan, UT, USA), 2 mM glutamine (Gibco, Grand Island, NY, USA), 10 ng/mL rEGF (Gibco, Grand Island, NY, USA), 5 mL of prepared insulin–transferrin–selenium reagent (Gibco, Grand Island, NY, USA) and 10,000 U/mL penicillin/streptomycin (Gibco, Grand Island, NY, USA).

The prepared mixture was seeded on a 3 cm diameter Petri dish, and the largest fragments were pressed to the bottom with a coverslip.

The cells were cultured in 5% CO_2_ at 37 °C, with the medium changed every two days. After 7 days of culturing, the coverslip was removed. Cells were passaged into a 25 cm^2^ plate vial at a density of 2 × 10^5^ cells/mL of medium in a volume of 5 mL after forming a confluent monolayer (after 14 days of cultivation).

The cells were counted using an automatic cell counter TC20 (Bio-Rad, Hercules, CA, USA). Their condition was evaluated at all stages of cultivation using a phase-contrast microscope (Olympus, Tokyo, Japan).

3. Cell characteristics.

The characteristics of the cultured cells, including their phenotype, proliferative activity and stemness, remained unchanged before and after transplantation into the collagen membrane.

The viability of the cells was determined using the vital dye of Calcein AM (ab14120, Abcam, Waltham, MA, USA) and Hoechst 33342 (Invitrogen, Carlsbad, CA, USA) for nuclear staining in fluorescence microscopy (Olympus, Tokyo, Japan).

Immunofluorescence analysis was used to determine the phenotype and level of proliferative activity of the cultured cells.

The P63 protein was determined as a marker of LSCs, and antibodies (ab735, Abcam, Waltham, MA, USA) at a dilution of 1:100 were used. Cytokeratin 3 and 12 (K3/12) were considered markers of mature keratocytes, and antibodies (orb4935, Biorbyt, Cambridge, UK) were used at a dilution of 1:250. Vimentin antibodies (ab24525, Abcam, Waltham, MA, USA) were used at a dilution of 1:750 to identify cells of mesenchymal origin (fibroblasts). Ki-67 staining (antibodies ab16667, Abcam, Waltham, MA, USA) was performed at a dilution of 1:200 to determine proliferative activity.

The immunofluorescence study was carried out as follows. Cells from the second passage were dispersed at a density of 4 × 10^3^ cells/mL in 96-well plates and on a collagen membrane; they were then cultured for 3 days, fixed in 4% paraformaldehyde for 20 min, washed three times with phosphate-buffered saline (PBS) and incubated in blocking buffer (2% fetal bovine serum + 0.5% Triton X-100 + 0.1% NaN3 in PBS). A primary antibody solution was applied to the cells in a blocking buffer. Incubation was carried out for 24 h at 4 °C. Afterward, the solution was poured out entirely, and the material was washed three times with PBS. After washing, the material was incubated for 40 min in the dark at room temperature in a secondary antibody solution in PBS: Alexa Fluor Plus 488 (donkey-anti-rabbit, 1:1000, Invitrogen, Carlsbad, CA, USA; A32790), Alexa Fluor 488 (goat-anti-chicken, 1:1000, Invitrogen, Carlsbad, CA, USA; A11093) or Alexa Fluor Plus 555 (donkey-anti-mouse, 1:1000, Invitrogen, Carlsbad CA, USA; A32773). The nuclei were stained with DAPI dye 40011 (Biotium, Fremont, CA, USA) at a dilution of 1:2000 for 10 min.

Images were captured using an inverted fluorescence microscope IX3-SSU (Olympus, Tokyo, Japan) equipped with a DP74 digital camera.

Cells from the second passage were used for further studies and seeded at a density of 3 × 10^4^ cells/cm^2^ on a 0.75 cm diameter collagen membrane composed of type I collagen with a concentration of 10 mg/mL.

### 2.3. Surgical Procedure

#### 2.3.1. Study of the Effect of Implantation of a Collagen Membrane (Carrier) in the Limbal Defect Area of Rabbits

The biocompatibility of the membrane in the experimental location—the limbal zone—was tested in the first stage of the study. A conjunctival incision was made along the limbus in the sector from 3 to 5 o’clock in the left eyes of two experimental rabbits for this purpose. As a control, the healthy (intact) right eyes of the same animals were used. The conjunctiva and tenon sheath were separated. Limbectomy was performed from 3 to 5 o’clock. The prepared Viscoll collagen membrane was then cut with scissors to the size and shape of the removed limbus and transplanted into the appropriate area. Three knotted sutures were placed on the cornea at 3, 4 and 5 o’clock (Neuron 10-0) to close the wound.

#### 2.3.2. Animal Model of LSCD

In 10 rabbits (10 eyes), a model of LSCD was obtained by mechanically removing half of the limbal tissue of the left eye. Healthy paired eyes were used as controls. An incision in the conjunctiva of the left eye along the limbus was performed from 8 o’clock to 2 o’clock. The conjunctiva and tenon sheath were separated, and hemostasis was performed. The limbal biopsy was removed using a sharp ophthalmic blade at a 45-degree angle, 1 mm from the corneal side and conjunctival side. Two knotted sutures were placed on the conjunctiva at 9 and 3 o’clock (Neuron 10-0).

The removed tissues were cut into 2 × 4 mm pieces and used as a biopsy for further cell culture.

To ensure the success of the LSCD modeling, one week after limbectomy, we additionally performed a chemical burn in the operation zone with 1 M NaOH, soaked the affected limbus with a sterile cotton swab for 30 s and then washed the ocular surface with PBS for 20 min.

To evaluate the effectiveness of LSCD modeling, the following typical features of the ocular surface were considered: erosion, corneal opacity and corneal neovascularization. Furthermore, goblet cells in the central zone of the cornea were found by impression cytology, which was absent in the healthy cornea.

#### 2.3.3. Group Formation

One month following the LSCD modeling, the animals were divided into two groups. The experimental group received tissue-engineered construct transplantation, while the control group only received conservative treatment: eye drops of dexamethasone 0.1% and levofloxacin 0.5% in one drop three times a day for 2 weeks. All ten rabbits had their corneal epithelium completely scraped with a scraper before treatment in order to measure the regeneration of the corneal epithelium.

#### 2.3.4. Transplantation Procedure

**Figure 1:**
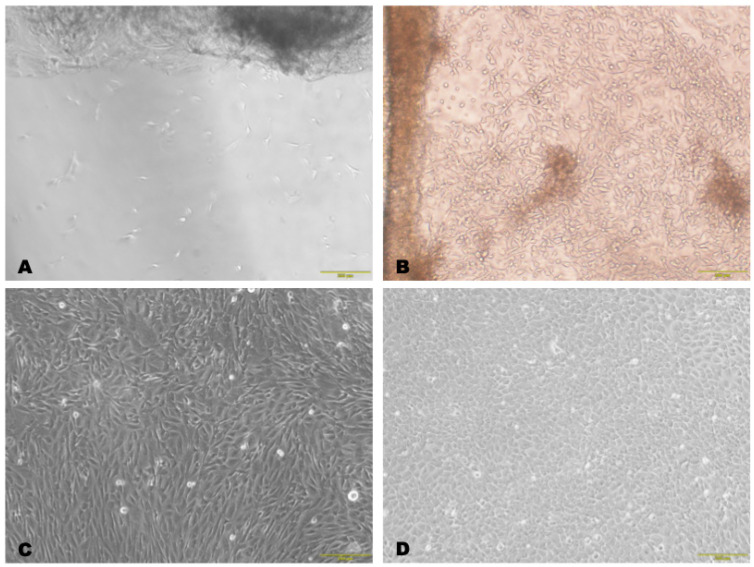
Photomicrographs of the primary cell culture from the limbal biopsy under a phase-contrast microscope: (**A**) single cells at the bottom of a Petri dish 3 days after sowing; (**B**) colonies of cells with polymorphic shape after 7 days; (**C**) loose monolayer with a mixture of epithelial-type cells and fibroblast-like cells after 10 days of cultivation; (**D**) dense subconfluent cell layer after 14 days of cultivation. The cells had predominantly polygonal shapes. Scale bar: 200 μm.

Under an operating microscope, a conjunctival incision was created in each of the five rabbits in the experimental group. The conjunctiva and Tenon capsules were separated. Abnormal fibrovascular tissue under the conjunctiva was removed using a microsurgical blade. Afterward, a scraper-assisted superficial keratectomy was carried out. Following the suitable form of the excised limbus, the tissue-engineered construct was cut using scissors and placed over the exposed surface of the removed limbal zone (Figure 1).

Four knotted sutures (Nylon 10-0) were positioned under the sclera to hold the graft in place and reduce cellular damage during surgery. The surface of the graft was covered with conjunctiva. In the end, two knotted sutures (Silk 8-0) were applied to the conjunctiva. Suture knots were rated and buried in the recipient stroma.

### 2.4. Postoperative Care

All rabbits were given anti-inflammatory therapy during the postoperative period, which consisted of subconjunctival injection with 0.5 mL solution of 0.4% dexamethasone once and three times daily instillation of 0.5% levofloxacin and 0.1% dexamethasone for 2 weeks.

### 2.5. Postoperative Examinations

Biomicroscopy with photo-registration was performed before treatment and on the third, seventh and thirtieth days, after which the clinical picture was assessed, including epithelial integrity, corneal transparency and the inflammatory reaction of the ocular surface. Slit-lamp biomicroscopy with fluorescent staining and anterior segment optical coherence tomography (AS-OCT) were performed.

### 2.6. Impression Cytology of the Ocular Surface

Furthermore, impression cytology with mucin staining was performed to detect goblet cells on the corneal surface. The technique was as follows: under topical anesthesia with Alcaine (proxymethacaine) 0.5%, the upper and lower eyelids were withdrawn. A 3 × 5 mm filter paper strip (MF-Millipore, Darmstadt, Germany) with a pore size of 0.22 μm was applied to the parts of interest on the ocular surface (central part of the cornea, limbal area or conjunctiva) and gently pressed with a glass rod for 5 s. Afterward, the strips were transferred to adhesive-coated slides, and the print was allowed to air-dry for thirty minutes before being fixed in absolute alcohol [20]. PAS-Alcian blue staining was performed according to the manufacturer’s protocol (Labiko, Saint-Petersburg, Russia). Subsequently, the preparations were observed with a Leica DM 2500 light microscope with photo-registration after being covered with a coverslip.

### 2.7. Histological and Immunohistochemical Analysis

Animals were euthanized by air embolism under general anesthesia for histological and immunohistochemical examination at 30 and 120 days. The eyes were enucleated. The corneoscleral flap was separated from the eyeball using a sharp ophthalmic blade. The flap was split in half; one half was fixed in 10% neutral formalin solution, and from this half, paraffin sections were subsequently prepared; the other half was fixed in 4% paraformaldehyde (PFA) solution and used for immunofluorescence investigations. The fixation process lasted 24 h at 4 °C.

Hematoxylin–eosin (H&E) staining was used to reveal the corneal morphology and corneal epithelium recovery on paraffin sections with a thickness of 8 μm, prepared according to the standard technique using xylene with alcohol. Histological specimens, stained with H&E, were examined under a Leica microscope.

Frozen tissues, which had been fixed in PFA, were sectioned to a thickness of 8 μm using a cryostat (Leica, Nussloch, Germany) for immunofluorescence analysis.

## 3. Results

### 3.1. Series of Experiments In Vitro

#### 3.1.1. Isolation of LSCs from Biopsy Specimens

Cell adhesion to the bottom of the cup was observed three days after cell isolation from a small-sized limbal biopsy using a modified protocol (Figure 1A). After seven days, colonies of epithelial-type cells filled the surface to approximately 40% confluency (Figure 1B). The obtained cells formed a loose monolayer after 10 days of culture and a dense subconfluent layer after 12 days (Figure 1C); then, at 14 days of culture, they were transplanted into a 25 cm^2^ plate (Figure 1D).

#### 3.1.2. Characterization of Isolated Cells

**Figure 2:**
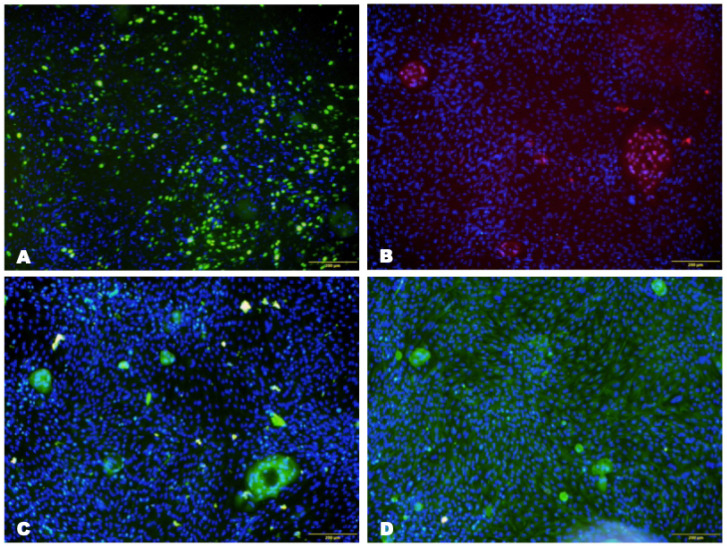
Photomicrographs of the cell culture in the plate from the second passage at 14 days of cultivation under a fluorescent microscope. Nuclei are stained with DAPI (blue). (**A**) Expression of Ki-67 (green), scale bar: 100 μm; (**B**) expression of P63 (pink), scale bar: 200 μm; (**C**) expression of CK-3/12 (green), scale bar: 200 μm; (**D**) expression of vimentin (green), scale bar: 100 μm.

The obtained limbal cells expressed CK3/12 and vimentin, indicating the presence of LSCs in the isolated cell population. In addition, the culture contained a high density of proliferating cells (22.9 ± 1.8%) that were stained positively for the Ki67 protein (Figure 2).

#### 3.1.3. Characterization of Cells on the Scaffold

**Figure 3:**
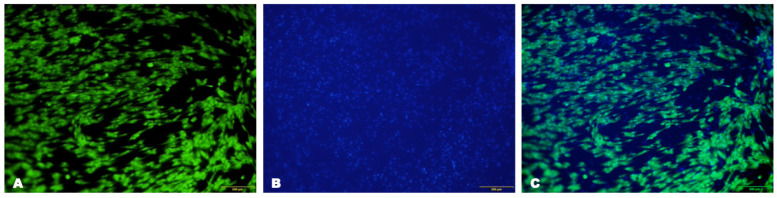
Photomicrographs of the cell culture on the collagen membrane from the second passage at 21 days of cultivation under a fluorescent microscope. (**A**) Calcein AM (green) was used to stain live cells, while (**B**) Hoechst (blue) was used to stain nuclei. (**C**) Merge and scale bar: 200 μm.

**Figure 4:**
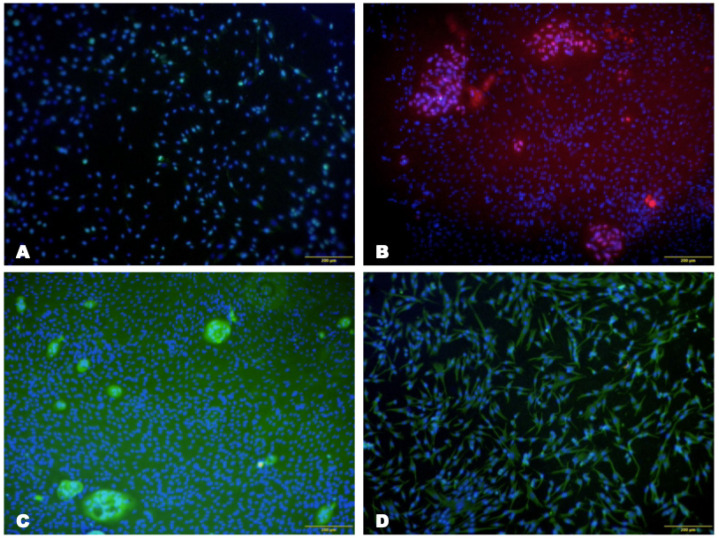
Photomicrographs of the cell culture on the collagen membrane from the second passage at 21 days of cultivation under a fluorescent microscope. Nuclei are stained with DAPI (blue). (**A**) Expression of Ki-67 (green), scale bar: 100 μm; (**B**) expression of P63 (pink), scale bar: 200 μm; (**C**) expression of CK-3/12 (green), scale bar: 200 μm; (**D**) expression of vimentin (green), scale bar: 100 μm.

The survival rate of LSCs on the collagen membrane was 89.6 ± 4.4% after seven days of culture (Figure 3). Furthermore, the expression of CK3/12 and vimentin was maintained in LSCs cultured on the collagen hydrogel matrix for seven days. The number of proliferating cells at seven days reached 27.7 ± 1.9% (Figure 4).

### 3.2. Series of Experiments In Vivo

#### 3.2.1. Evaluation of Local Tissue Response to Collagen Hydrogel Implantation into the Limbal Defect Area of Rabbits

**Figure 5:**
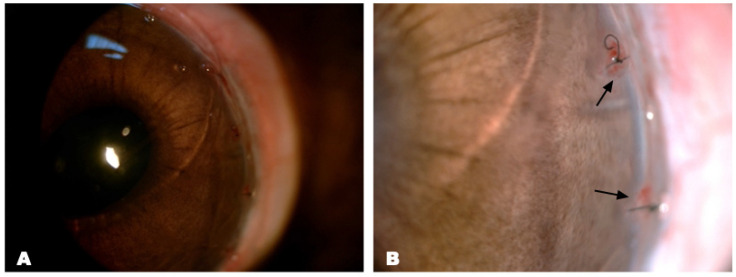
Biomicroscopic images of the eye after limbectomy and transplantation with collagen membrane within a month. (**A**) The cornea is transparent, with a smooth surface and no signs of inflammatory infiltration. The star represents the collagen membrane implant. (**B**) Local reaction from the nodular suture with ingrowth of vessels (arrows).

**Figure 6:**
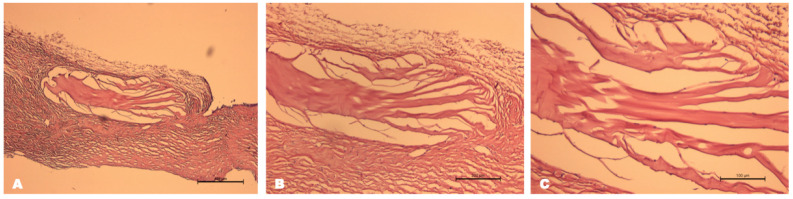
Histological section of limbal area with the Viscoll collagen membrane. In the thickness of the collagen implant, single migrated cells with hyperchromic nuclei were observed. Stain: hematoxylin and eosin. (**A**) Scale bar: 400 μm; (**B**) scale bar: 200 μm; (**C**) scale bar: 100 μm.

The cornea remained transparent after limbectomy with collagen hydrogel implantation, and there were no signs of immune rejection, such as corneal edema, epithelial defects or infiltration. A small area of focal reaction in the form of blood vessel ingrowth was detected at the site of the knotted sutures (Figure 5). Histologic examination of the enucleated eye was performed one month after the operation. The histologic section revealed a collagen membrane that was covered by conjunctival epithelium. In addition, the migration of single cells with large hyperchromic nuclei inside the implant was detected (Figure 6A). No signs of inflammation, leukocyte infiltration or fibrosis were observed (Figure 6B). As a result, it can be concluded that the collagen biomaterial used is biocompatible and does not cause a toxic reaction when transplanted into the limbal zone.

#### 3.2.2. LSCD Modeling

##### Clinical Manifestations (Slit-Lamp Biomicroscopy with Photo-Registration)

**Figure 7:**
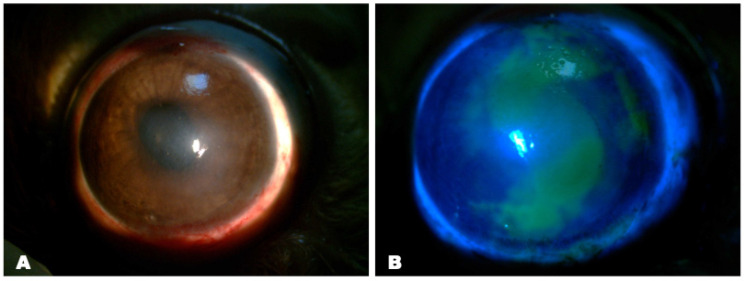
Biomicroscopic images after modeling of LSCD. (**A**) Cornea slightly edematous erosion in the center with superficial opacity. Neovascularization from limbus to corneal periphery. (**B**) Corneal erosions were stained with fluorescein (green).

In all experimental animals, after the mechanical removal of half of the limbal tissue and subsequent chemical burning within three days, there was an increase in inflammatory reactions and corneal edema with peripheral opacity. During the first week, the inflammation and swelling subsided gradually, with blood vessels growing into the limbal area. By the end of one month, partial or full clinical signs of LSCD were revealed, which included corneal neovascularization, opacity, surface irregularities and persistent corneal erosion (Figure 7).

##### Impression Cytology

**Figure 8:**
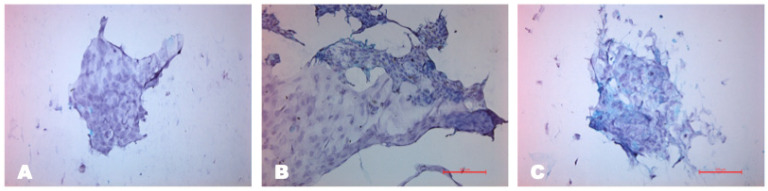
Impression cytology in different anatomical areas of the eye after LSCD modeling with PAS staining, scale bar: 100 μm. (**A**) Conjunctival epithelial cells with high nuclear/cytoplasmic ratio; (**B**) in the limbal area, borderline between conjunctival and corneal epithelium; (**C**) blue periodic acid–Schiff-positive goblet cells stained with mucin were observed in the central cornea.

According to the results of impression cytology, 30 days after LSCD modeling, the conjunctivalization of the central and peripheral parts of the corneal epithelium was revealed. The presence of goblet cells was noted in these zones, which were absent in the healthy eye. This is the most reliable diagnostic criterion for the diagnosis of LSCD, which indicates a successful animal model (Figure 8).

##### AS-OCT

**Figure 9:**
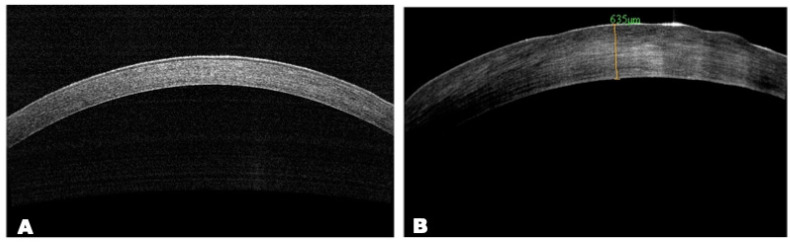
AS-OCT images. Scans of central cornea: (**A**) normal cornea; (**B**) AS-OCT scan shows area of anterior corneal haze with hyperreflective zone and epithelial defects; optical section is thickened due to corneal edema.

AS-OCT data showed the presence of epithelial irregularity and stromal thinning with a hyperreflective area in the central zone of the cornea (Figure 9).

#### 3.2.3. Epithelial Regeneration after Matrix-Assisted Cell Transplantation

##### Clinical Manifestations (Biomicroscopy with Photo-Registration and Fluorescent Staining)

Experimental animals from the experimental group (matrix-assisted cell transplantation) and control group (instillation of 0.1% dexamethasone and 0.5% levofloxacin) were observed for a month after treatment. In both groups, positive dynamics were noted as a reduction in the size of corneal erosion.

**Figure 10:**
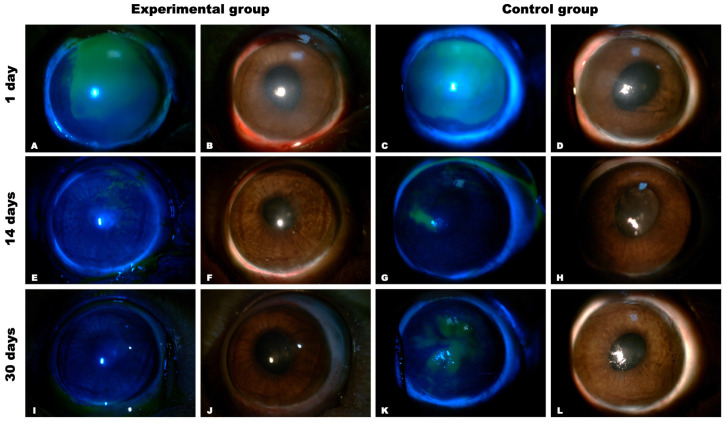
Clinical changes in the eyes after treatment by slit-lamp examination with and without fluorescent staining. The left two panels (rows) of images show specimens after matrix-assisted cell transplantation, while the right two panels (rows) show reconstructed ocular surfaces after conservative treatment. Fluorescein staining is green, which represents epithelial defects. Corneal opacity and edema were significantly decreased in the experimental group with matrix-assisted cell transplantation (**B**,**F**,**J**); at 30 days, there was no corneal fluorescein staining (**A**,**E**,**I**); corneal opacity and edema were decreased in the control group with instillation of anti-inflammatory eye drops, but, at 30 days, corneal epithelial irregularity was observed (**D**,**H**,**L**); the green fluorescence persisted in the corneas at the end of 30 days with decreased area (**C**,**G**,**K**).

In both groups, there was a noticeable thickening of the corneal stroma with edema during the first 48 h following surgery, giving poor visibility to the iris and pupil (Figure 10).

After 14 days of therapy, in the experimental group, corneal epithelization was nearly fully restored, whereas the areas with erosion persisted in the control group.

After a month, the experimental group showed full corneal epithelization and almost total restoration of corneal transparency while maintaining a slight degree of superficial opacity (Figure 10). Simultaneously, there was epithelial irregularity and corneal erosion with more noticeable opacity in the controls (Figure 10).

##### Impression Cytology

According to the data of impression cytology, 30 days after transplantation in the experimental group, epithelial cells in the central area of the cornea showed a normal shape, and goblet cells were absent. Furthermore, the limbalization of the conjunctiva was observed, with a similar shape to cells from the limbal zone, indicating the migration and viability of transplanted cells in two directions, towards the cornea and conjunctiva.

**Figure 11:**
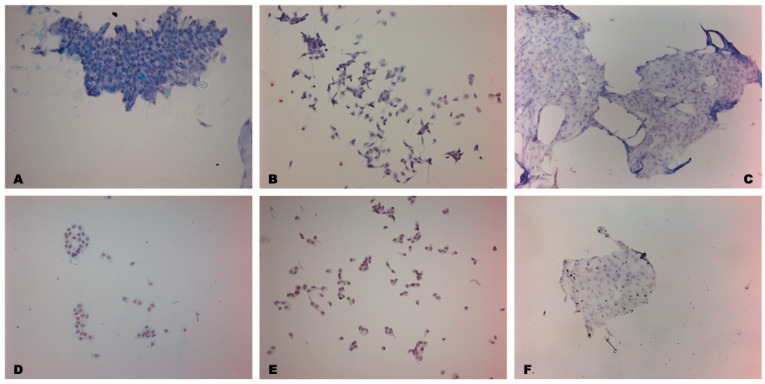
Impression cytology in different anatomical areas of the eye after treatment with PAS staining, scale bar: 100 μm. Upper panels show results in the control group; lower panels show the experimental group. (**A**) Conjunctival epithelial cells remain unchanged, with small nuclei and good cell-to-cell adhesion. (**B**) In the limbal area, the cells are small in shape, reflecting conjunctival epithelial cells. Goblet cells are present in this area. (**C**) Corneal epithelial cells are altered in the shape of the nucleus, with elongated polymorphic shapes in the central cornea. Blue periodic acid–Schiff-positive goblet cells stained with mucin are observed. (**D**) Analogous limbal cells in the conjunctiva are observed with small nuclei and high nuclear/cytoplasmic ratio, which indicates limbalization in this area. (**E**) Small round cells in the limbal zone with polymorphic nuclei. (**F**) Goblet cells are absent in the cornea, and polygonal corneal epithelial cells with normal shape are restored.

At the same time point, goblet cells persisted in the central part of the cornea in samples from the control group, indicating that corneal conjunctivalization was preserved. In addition, cell metaplasia was observed in the limbal zone, manifested as a change in the shape of cells with polymorphic nuclei and a large nuclear/cytoplasmic ratio. The number of metaplasized cells in the control group was higher than in the experimental group (Figure 11).

##### AS-OCT

Data from the AS-OCT 30 days after treatment showed the restoration of corneal contours with the preservation of slight superficial opacity in the experimental group. The epithelialization was complete, and the corneal surface was smooth.

**Figure 12:**
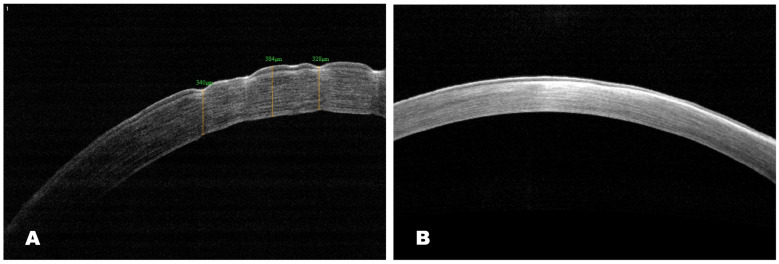
AS-OCT images. Scans of central cornea: (**A**) scan shows epithelial defects with corneal irregularity, subepithelial corneal opacity with hyperreflective areas in the control group; (**B**) AS-OCT scan shows restoration of corneal epithelial integrity with superficial haze in the experimental group.

In the same period, the corneal surface was rough and slightly thickened, with the preservation of erosion and edema (Figure 12). The results matched the biomicroscopic picture.

##### Histological Examination

Histological examination was performed 90 days after treatment. In the experimental group, the corneal stroma was covered with a multilayer stratified epithelium consisting of 4–5 layers with large nuclei in the histological sections of the central cornea; no goblet cells were found in these sections. On the basal layer, vacuoles and round nuclei were observed inside the basal cells. The middle layers showed cells with a regular prismatic morphology, and the upper layer was formed by flattened superficial cells with elongated nuclei.

**Figure 13:**
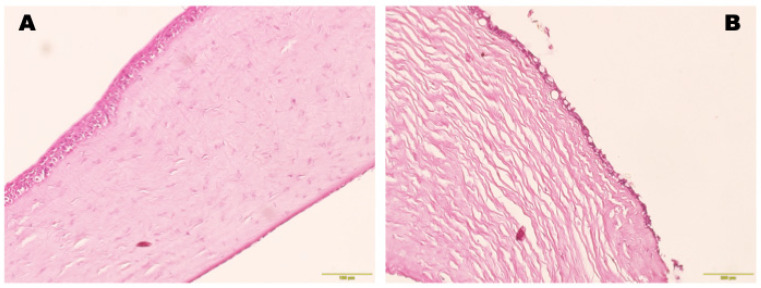
Photomicrographs of the histological cornea section after treatment. Stain: hematoxylin and eosin. (**A**) Histological section of the cornea shows restoration of the corneal epithelium with 3–5 layers of stratified epithelial cells in the experimental group; no goblet cells were found in the sections. Scale bar: 100 μm. (**B**) In the control group, goblet cells were detected in the histological sections of the central cornea, but the corneal architecture was altered. The histological corneal sections showed a multilayer stratified epithelium consisting of 2–4 loosely arranged layers. Scale bar: 200 μm.

In the control group, goblet cells were detected in the histological sections of the central cornea, but the corneal architecture was altered. The histological corneal sections showed a multilayer stratified epithelium consisting of 2–4 loosely arranged layers. Basal cells had prismatic shapes and more vertically elongated, irregular nuclei. Conjunctival metaplasia in the corneal epithelium was detected more frequently in the control group. There was no evidence of inflammatory cell infiltration in the extracellular matrix (Figure 13).

##### Immunohistochemical Analysis

**Figure 14:**
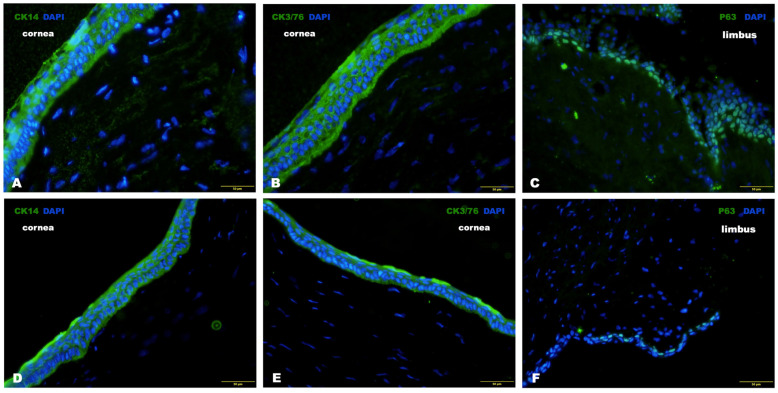
Immunofluorescence staining of CK14, CK3/76, P63 in the central cornea and limbus after treatment: (**A**–**C**) results in the experimental group; (**D**–**F**) the control group.

The immunohistochemical results showed the expression of CK14, CK3/76 and P63 in both groups, indicating corneal epithelium regeneration with the restoration of differentiated corneal epithelial cells in the central corneal zone and the presence of LSCs in the limbal area. Moreover, in the experimental group, the number of these cells was noticeably higher than in the control group (Figure 14).

## 4. Discussion

Limbal stem cell deficiency is a disorder of the ocular surface leading to the progressive loss of corneal transparency and visual impairment. The situation is complicated by the fact that the existing surgical treatment methods associated with the transplantation of a donor cornea cannot ensure long-term transparent engraftment. This forces surgeons to perform additional manipulations to achieve limbal zone reconstruction. However, due to limited donor material and immunologic reactions related to tissue compatibility, this problem can only be partially solved.

Cell-based therapy holds great promise for the treatment of LSCD. Clinical and experimental data suggest that the use of LSCs to treat LSCD has the potential to revolutionize the ophthalmology field [21]. However, there are numerous barriers that have slowed the development and adoption of cell therapy in clinical practice [22]. These challenges include, for example, the choice of the cell delivery method to ensure optimal efficacy, as poor cell engraftment and poor survival after transplantation are serious problems.

The transplantation of such cells as part of the matrix improves survival and integration with the surrounding tissues [23,24].

There are various protocols for the culture of LSCs for transplantation, including cell dissociation methods from a biopsy (mechanical dissociation or enzymatic digestion) and culture and expansion on various carriers (amniotic membrane, fibrin or biomaterials with collagen) [25,26]. We used a modified enzymatic digestion protocol in combination with explant culture to ensure the faster acquisition of LSCs and limbal niche cells, which can potentially influence the treatment efficiency by allowing one to perform the surgical intervention in a short period of time.

Both synthetic and natural polymers are used as carriers. In 2019, Karpovich V.V. and colleagues published a comparative analysis of the properties of synthetic polyester matrices composed of polylactide–glycolide, polylactide–caprolactone and poly-ε-caprolactone. The authors found that a five-micron-thick polylactide–caprolactone matrix showed higher transparency, strength and biocompatibility of cultured cells, and similar properties to an amniotic membrane [27]. In another study, the authors used a neutral cross-linked polymer (end-linked PEG (poly(ethylene glycol))) for implantation into the corneal stroma. However, the experimental rabbit developed side effects within three months after the implantation of the matrix based on PEG-diacrylate, including an inflammatory reaction, ulceration and diffuse corneal opacity [28].

Natural materials have significant advantages over synthetic ones due to their high biocompatibility. However, they are difficult to manufacture and have poor biomechanical properties. Collagen is a structural protein in the fibrous layer of the eyeball and the main component of the extracellular matrix; thus, it is structurally as close to the cornea and limbus as possible, which was the primary reason for the use of this carrier in our study.

Type I collagen is biodegradable and contains cell-adhesive sequences required for cell migration, proliferation and survival. In comparison to other carriers, collagen is the most preferable because it allows for the creation of stable (collagen is resistant to most enzymes, unlike fibrin) and standardizable biomaterials. The disadvantages of collagen biomaterials are generally regarded as inadequate mechanical properties for standard surgical manipulations. However, we recently demonstrated that the use of higher concentrations of collagen (more than 10 mg/mL) can overcome this problem [18].

Furthermore, several scientists have demonstrated that the use of LSCs as part of a collagen-based material allows for the creation of a depot for corneal epithelium regeneration. In one study, the authors used gelatin composed of type I collagen with a low concentration (2 mg/mL), which biodegraded quickly and was unable to maintain an optimal environment for cultured LSCs for a long time. In addition, there was no evaluation of the phenotypic and proliferative characteristics of cells after culture in this medium. Therefore, in our work, we attempted to eliminate these limitations. In our study, we attempted to use native type I collagen that had not been chemically modified as a matrix for cell transfer to the limbal zone, ensuring maximum biocompatibility. To address the issue of rapid biodegradation, we used a high concentration (20 mg/mL). It was described by Chae J. J. and his colleagues that a collagen Vitrigel membrane (CVM) with a low concentration of type I collagen (0.5%) can be used as a carrier for cultured LSCs to treat total LSCD resulting from a chemical burn [29]. Despite the positive results, the rapid resorption of the material and difficulty with surgical manipulations were noted.

Based on data from the slit-lamp examination and histology, we concluded that the investigated collagen carrier did not cause toxic or inflammatory reactions when transplanted into the limbal zone and can be used as a carrier for cultured LSCs and further transplantation into the body.

An important point in our study is the technological possibility of obtaining a sufficient number of cells for further culture and transplantation from a small biopsy specimen of 2 mm in size; such a small size will not cause iatrogenic LSCD in the healthy eye and makes the technique closer to the real application in the practice of ophthalmology [30]. In comparison to other methods, this allows for a reduction in the risk of cell damage and the reliable fixation of the graft [31,32].

To ensure higher proliferative activity, the stemness of cultured cells and the possibility of transplantation, we used cells from the first passages.

With regard to stem cell therapy, demonstrating the presence, survival and availability of stem cells both in culture and as part of the medium is critical for procedure validation. In this study, we demonstrated that the cultured cells on the collagen matrix were a mixture of LSCs and MSCs with high proliferative activity and survival rates, stained positively with markers P63 and vimentin, which maintained a survival rate of approximately 90%.

More importantly, the transplantation of this biomaterial into the damaged limbus results in the active regeneration of the corneal epithelium, which indicates not only the survival of viable cells in the implanted matrix after transplantation but also their functional activity.

Considering that the rabbit cornea tends to self-repair without therapy [33], we demonstrated the success of modeling partial LSCD in rabbits. This was confirmed by the typical clinical manifestations observed by biomicroscopy and the presence of goblet cells in the central zone of the cornea observed by impression cytology. One month after treatment, all clinical signs of LSCD were preserved in the control group, whereas the active regeneration of the corneal epithelium was revealed in the experimental group. According to the histological analysis 90 days after treatment, in the control group, goblet cells were found in the central corneal epithelium, which contained 2–4 loosely arranged layers of epithelial cells. In the experimental group, the stroma was covered with a multilayer stratified epithelium without goblet cells in the central epithelium. Furthermore, the restoration of the tissue architecture of the epithelium was revealed.

In both groups, immunohistochemical examination of the animals’ corneas revealed the expression of CK14, CK3/76 and P63. Moreover, the number of these cells in the experimental group was higher than in the control group.

The criteria for the evaluation of treatment effectiveness consist of the restoration of the anatomical integrity of the epithelium and corneal transparency in experimental animals. The lack of uniform standardization of treatment efficacy assessment makes the comparison of different methods and efficacy in different studies difficult. The majority of studies do not include impression cytology or AS-OCT in combination with standard slit-lamp biomicroscopy as research methods. Only the latter is used for observation to assess the clinical signs. In some studies, the success of LSCD modeling before treatment has not been demonstrated. Therefore, in our study, biomicroscopy with photo-registration and fluorescent staining, AS-OCT and impression cytology was performed on rabbits for the diagnosis of LSCD.

The results obtained correspond to the results obtained in the study conducted by Chae J. J. They used collagen Vitrigel, which contained a low concentration of collagen 0.5%, as a carrier for cultured LSCs in the treatment of previously created total LSCD by chemical burns. The researchers discovered that the transplantation of a tissue-engineered construct of CVM and cultured LSCs was effective in treating LSCD, which suggests that type I collagen is helpful in regenerating the epithelium and maintaining corneal epithelial differentiation.

In comparison to the conservative method of treatment, we found that cell-assisted matrix transplantation consisting of a cultured autologous population of LSCs and a collagen carrier reduced the time of corneal defect healing, and it is an effective method for treatment as well as corneal epithelium restoration.

## 5. Conclusions

The matrix-assisted cell transplantation of LSCs with the support of a collagen membrane was presented in this study. It was shown that it is an effective method of treatment for LSCD in experimental animals. However, further observation and evaluation of the long-term results are required. The method may be recommended for further study with the aim of entering clinical practice.

## Data Availability

Data are contained within the article.
